# First person – Gonzalo Quiroga-Artigas

**DOI:** 10.1242/bio.060352

**Published:** 2024-02-27

## Abstract

First Person is a series of interviews with the first authors of a selection of papers published in Biology Open, helping researchers promote themselves alongside their papers. Gonzalo Quiroga-Artigas is first author on ‘ Storage cell proliferation during somatic growth establishes that tardigrades are not eutelic organisms’, published in BiO. Gonzalo is a postdoc in the lab of María Moriel-Carretero at Centre de Recherche en Biologie cellulaire de Montpellier (CRBM), Université de Montpellier, France, delving into the genetic factors that influence the extremophile abilities of tardigrades.


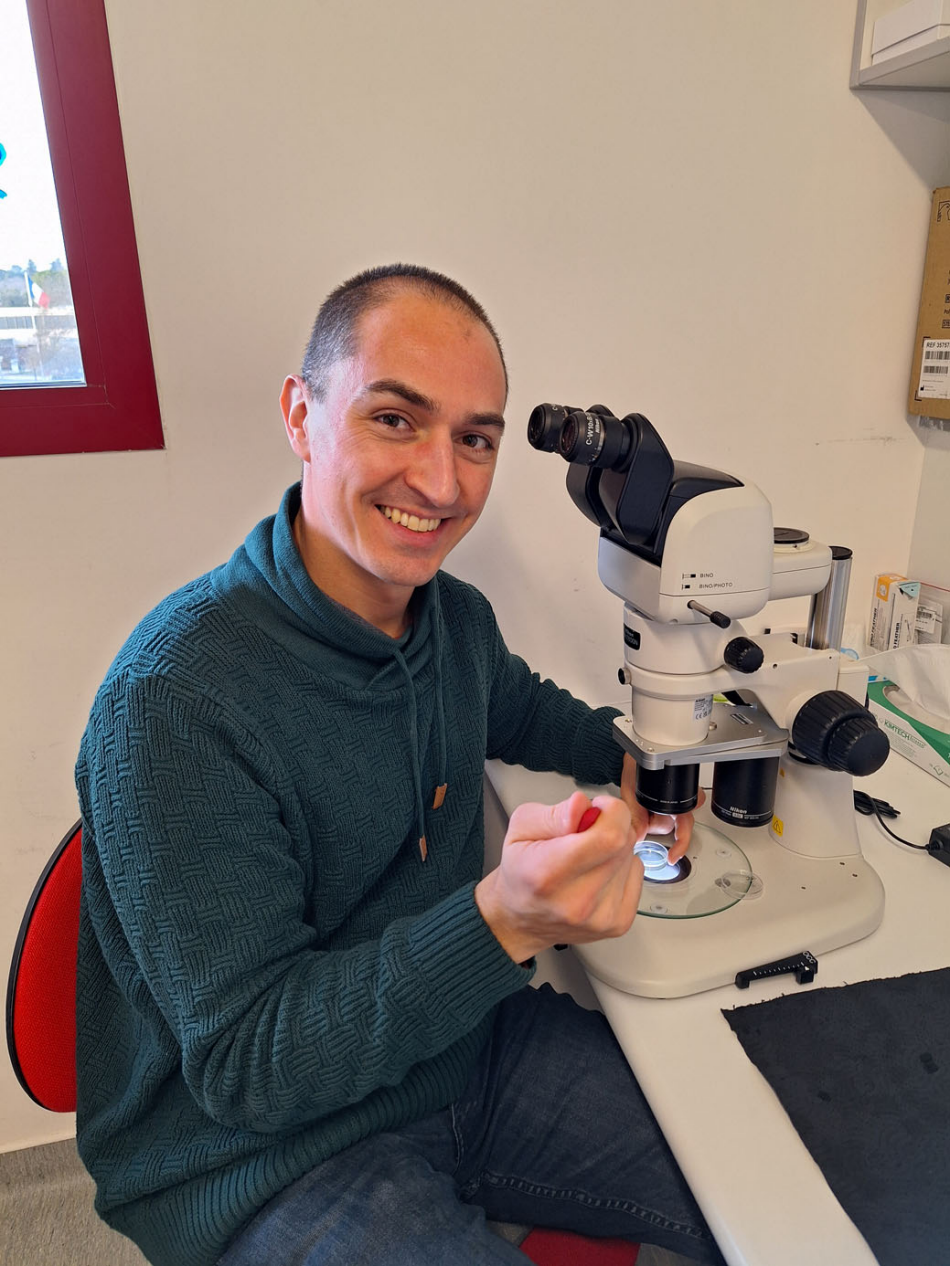


**Gonzalo Quiroga-Artigas**

**Describe your scientific journey and your current research focus**

After completing my bachelor's studies in biology and earning a master's degree in genetics and genomics from the University of Barcelona, I embarked on my PhD adventure in Nice, France, under the guidance of Dr Evelyn Houliston. My doctoral research, titled ‘Light-induced oocyte maturation in the hydrozoan *Clytia hemisphaerica*,’ was conducted as part of the Marie Curie ITN program NEPTUNE. This experience provided invaluable networking opportunities, fostering regular interactions with leading scientists in the field of evo-devo across Europe. I successfully obtained my PhD in biological sciences in May 2017. Continuing my academic journey, I initiated my postdoctoral research at The Whitney Laboratory for Marine Bioscience in the USA, working under the mentorship of Dr Christy Schintzler. During this phase, I focused on the study of stem cell-driven regeneration in the hydrozoan *Hydractinia symbiolongicarpus*. Currently, I am in the midst of my second postdoc at the Centre de Recherche en Biologie cellulaire de Montpellier (CRBM), under the supervision of Dr María Moriel-Carretero. In my current research, I am investigating the mechanisms of extremotolerance in tardigrades, utilizing the model *Hypsibius exemplaris*.

**Who or what inspired you to become a scientist?**

From a young age, I have been captivated by the beauty of natural environments. Whether exploring forests, snorkeling or scuba diving underwater, or even observing life in urban areas, my attention has always been drawn to the intricacies of living organisms. The influence of Sir David Attenborough and his BBC documentaries played a pivotal role in inspiring my decision to pursue a degree in biology. Once enrolled in university, my fascination for the natural world deepened. The relentless curiosity to comprehend biological processes fueled my desire to transition from a student of biology to a scientific researcher.

**How would you explain the main finding of your paper?**

Tardigrades, microscopic molting invertebrates renowned for their extreme environment resilience, were traditionally believed to maintain a constant cell number after completing embryonic development, a phenomenon termed eutely. Our paper presents molecular evidence supporting the proliferation of a specific tardigrade cell type known as ‘storage cells’ during the animal's growth, leading to an augmentation in their total cell numbers. This proliferation occurs in bursts synchronised with each molt of the cuticle. Additionally, my data underscore the crucial role of DNA replication, preceding cell proliferation, for tardigrade survival. This research provides a comprehensive insight into DNA replication events throughout tardigrade growth, and definitely establishes that tardigrades are not eutelic organisms.

**Figure:**
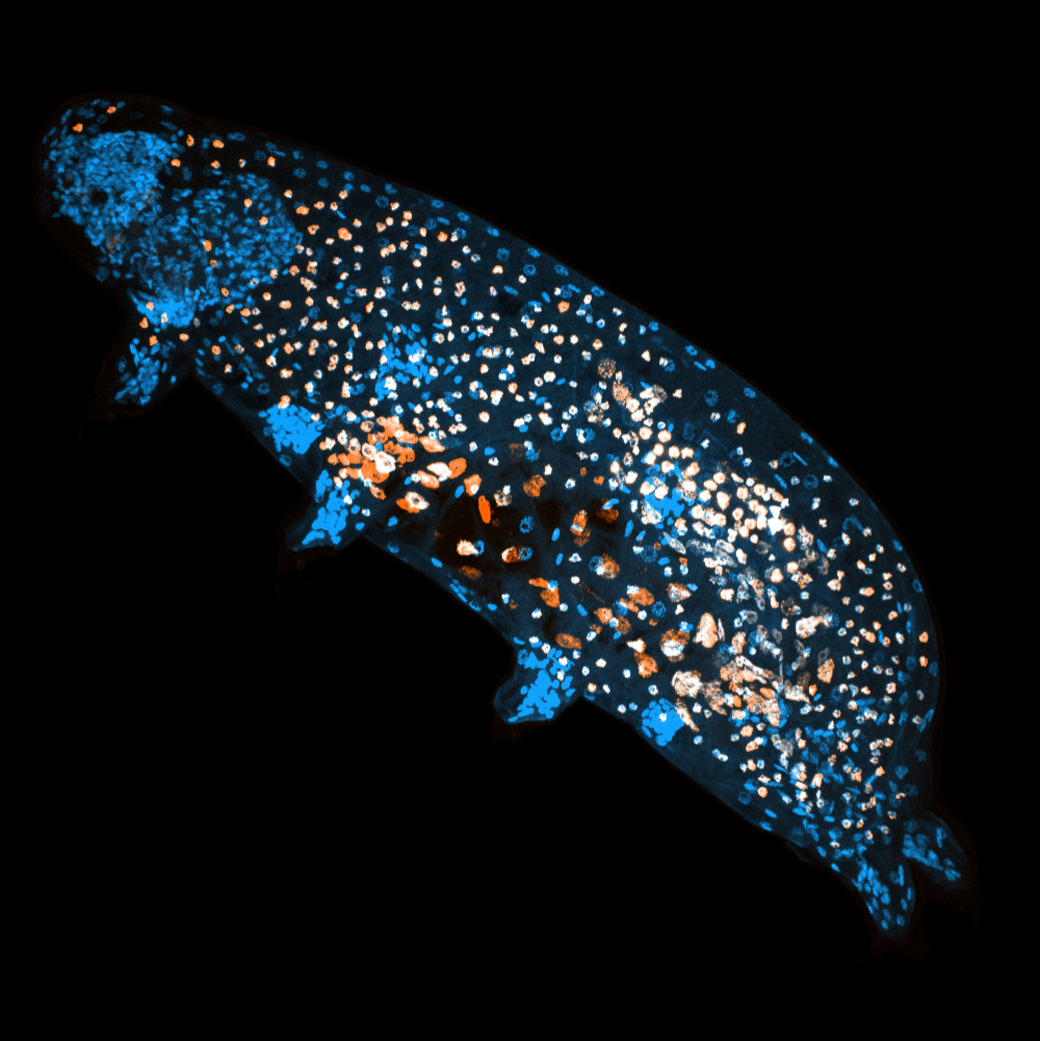
**The tardigrade *Hypsibius exemplaris* upon 3-week incubation in EdU.** Cell nuclei are displayed in blue, while EdU^+^ cells are shown in orange. This image highlights EdU incorporation in gut cells, germ cells, and storage cells, demonstrating cell proliferation and thus cell number increase during tardigrade somatic growth.

**What are the potential implications of this finding for your field of research?**

When I started to study tardigrades roughly a year and a half ago, I encountered a recurrent narrative in both peer-reviewed scientific literature and tardigrade biology outreach websites, characterizing tardigrades as eutelic animals (cell numbers constancy after embryonic development). Through my work, I hope to persuade the scientific community to refrain from categorizing these remarkable animals as ‘eutelic organisms’. Moreover, the finding that storage cells undergo replication and proliferation opens new possibilities for *in vitro* culture of this cell type, and positions it as a valuable model for studying cell cycle dynamics, responses to replication stress, and DNA damage management in tardigrades.

Through my work, I hope to persuade the scientific community to refrain from categorizing these remarkable animals as ‘eutelic organisms’.

**Which part of this research project was the most rewarding?**

When I started my second postdoc about a year and a half ago, we did not even have tardigrades in the lab, and neither my mentor nor I had prior experience working with these animals. However, within this relatively brief period, I successfully established the tardigrade *H. exemplaris* as a model in our lab and generated sufficient data to publish a sound scientific paper. This marks my inaugural publication employing tardigrades as an experimental model system, and the experience itself has proven to be highly rewarding for me.

**What piece of advice would you give to the next generation of researchers?**

For the next generation of researchers, my advice is to pursue a career in academia only if you have a genuine passion and vocation for it. While research in academia can be highly rewarding and a beautiful experience, it can also be a challenging path. Passion serves as a crucial anchor during tough times, making it easier to bounce back when things don't go as planned. Stay strong, persevere, and continue fighting for the career you love!

**What's next for you?**

I am hoping to secure a permanent researcher position within the Centre National de la Recherche Scientifique (CNRS) in France. My ambition is to advance my research on tardigrades within the lab of Dr María Moriel-Carretero, as our distinct expertise has already proven to be highly complementary. However, I am open to the possibility of leading my own research team in the future.
